# The association between psychosocial factors and change in lifestyle behaviour following lifestyle advice and information about cardiovascular disease risk

**DOI:** 10.1186/s12889-018-5655-7

**Published:** 2018-06-13

**Authors:** Rebecca A Dennison, Adina L Feldman, Juliet A Usher-Smith, Simon J Griffin

**Affiliations:** 1 Primary Care Unit, Department of Public Health and Primary Care, University of Cambridge, Cambridge, CB2 0SR UK; 2 MRC Epidemiology Unit, Institute of Metabolic Science, University of Cambridge, Cambridge, CB2 0SL UK

**Keywords:** Behaviour change, Cardiovascular disease, Fruit and vegetable intake, Physical activity, Psychosocial factor, Randomised controlled trial, Risk

## Abstract

**Background:**

Physical activity (PA) and fruit and vegetable intake (FVI) are two key modifiable risk factors for cardiovascular disease (CVD). Achieving change in these behaviours is challenging and affected by many variables including psychosocial factors. We aimed to investigate the association between social support, stress and mood, and change in PA and FVI following provision of CVD risk information and web-based lifestyle advice.

**Methods:**

Seven hundred sixteen blood donors (56% male; mean age 57 years) from the intervention arms of the Information and Risk Modification (INFORM) trial, a randomised controlled trial to assess the impact of providing CVD risk and web-based lifestyle information, were analysed as a prospective cohort. We used linear and logistic regression analyses to quantify the association between social support, stress and mood at baseline and behaviour change following the intervention. We modelled objective (average acceleration measured by Axivity AX3 wrist-worn accelerometers and plasma carotenoid levels) and subjective (self-reported recreational PA and FVI) outcomes as change between baseline and 12 weeks follow-up.

**Results:**

There was no clear association between social support and change in objective or subjective PA. Higher levels of stress and, to a lesser extent, depression symptoms were associated with smaller improvement in self-reported PA (β -1.53 h/week vigorous PA, 95% confidence interval (CI) -2.30 to -0.75, *p* < 0.001 for stress; β -1.64 h/week, 95% CI -3.50 to 0.21, *p* = 0.082 for little interest). Higher social support was associated with greater odds and higher stress was associated with lower odds of increasing self-reported FVI to five portions per day (odds ratio (OR) 1.33, 95% CI 1.05 to 1.69, *p* = 0.020 for social support; OR 0.57, 95% CI 0.43 to 0.76, *p* < 0.001 for stress). The associations between psychosocial factors and objective FVI were not statistically significant.

**Conclusions:**

High stress and low mood may reduce the likelihood and extent of reported change in PA and FVI following CVD risk information and advice. Greater social support may be associated with increased FVI. The role of psychosocial factors should be considered when developing, tailoring and evaluating future interventions.

**Trial registration:**

Current Controlled Trials ISRCTN17721237. Registered 12 January 2015.

**Electronic supplementary material:**

The online version of this article (10.1186/s12889-018-5655-7) contains supplementary material, which is available to authorized users.

## Background

Cardiovascular disease (CVD) is the leading global cause of mortality and morbidity, causing an estimated 17.7 million (31%) deaths in 2015 [1]. Risk of CVD is influenced by many aspects of lifestyle including diet and physical activity (PA). There is evidence that populations are eating less healthy diets and becoming more sedentary [2]. Small lifestyle changes in large numbers of people have the potential to reduce the incidence of CVD risk in the population, consequently promoting behaviour change is a public health priority. Provision of CVD risk information and lifestyle advice is advocated by international guidelines and has been offered to people aged 40 to 74 years in England since 2009 through the National Health Service Health Checks programme [3, 4]. However, while provision of risk information has been shown to increase the accuracy of CVD risk perception, there is no evidence that it reduces CVD incidence [5].

There is, however, strong evidence for the health benefits of PA and high fruit and vegetable intake (FVI), including lower risks of premature mortality and CVD [6, 7]. Despite this, recent Health Survey for England data show that only 5% of individuals meet the national PA guidelines in the UK (measured by accelerometers; when self-reported PA is assessed, this is closer to 40%) and only a quarter of people report that they meet the target consumption of five portions of fruit and vegetables per day (5 A Day) [8]. Changes have not been observed in this population over recent years [9], therefore increasing PA and FVI remains a priority in reducing the risk of CVD.

Sustained changes in behaviour are difficult to achieve. As demonstrated in models such as the behaviour change wheel [10], behaviour is subject to a range of influences including psychosocial factors. Social support describes the supportive behaviours provided by other people and the relationships that one experiences [11]. It has been found to correlate with levels of PA in both men and women and across different types and intensities of PA [12]. Anderson-Bill reported that participants in a web-based intervention with support from their friends and family were more likely to set goals and monitor their behaviour [13]. Similarly, relatives of CVD patients with greater social support had greater adherence to interventions to promote PA and healthier diet [14, 15]. Conversely, other studies have found no clear positive association between social support and change in PA following interventions, with either subjective [16] or objective measures of outcome [17]. One literature review reported that social support predicted FVI in adults [18] whereas a similar review that only included studies based on social cognitive theories did not find this association [19].

There is also considerable evidence to suggest that higher levels of stress are associated with less exercise and PA, and also that PA can reduce stress [20]. Conversely, two reviews assessing the association between psychosocial factors and FVI have not reported an association with stress [18, 19], although less healthy foods are consumed when stressed [21], and sugary and fatty foods can alleviate stress in some circumstances [22].

Similarly, there is a bidirectional inverse association between low mood and exercise: less PA is associated with a higher likelihood of depressive symptoms [23] and depression is consistently associated with declining levels of PA [24]. While the association between depression and FVI is perhaps less consistent and well documented, evidence suggests that FVI is inversely related to risk of depression [25] and is lower in people with depression [26]. In addition, there is some evidence that participants with depression are less likely to initiate or sustain a lifestyle behaviour change regime [27].

Evidence for the role of social support, stress and experience of low mood in determining levels of PA and FVI therefore suggests that they could influence the intention or ability to change lifestyle behaviour. However, the effect of these psychosocial factors on change in lifestyle behaviours following provision of risk information and lifestyle advice is inconclusive. We aimed to assess the association between psychosocial factors (social support, stress and mood) and short-term change in objectively measured and self-reported PA and FVI following provision of CVD risk and lifestyle behavioural change information.

## Methods

### The INFORM trial

The INFORM (Information and Risk Modification) trial was a parallel-group, open randomised controlled trial in England that sought to quantify the short term effects of providing information about CVD risk and lifestyle advice on health-related behaviours; the protocol has been reported previously [28]. A convenience sample of 932 participants recruited from the INTERVAL blood donation trial [29] was randomised 1:1:1:1, with stratification by age (older or younger than 60 years) and sex, to one of the following trial arms: control with no intervention; web-based lifestyle advice only; lifestyle advice plus phenotypic coronary heart disease (CHD) risk score information; or lifestyle advice plus phenotypic and genetic CHD risk score information. For this analysis, the control group was excluded in order to assess the association between psychosocial factors and behaviour change in people who received the intervention.

### Exposures and outcomes

Social support was measured as part of a baseline questionnaire using the previously validated 12-item Multidimensional Scale of Perceived Social Support (MSPSS) that covers three dimensions: social support from family, friends and a significant other [30]. We excluded participants if more than half the responses from one of the dimensions were missing and calculated a mean score between one and seven; higher scores indicated greater perceived social support. Participants were also asked, “How would you rate your overall stress level?” on a five-item Likert scale from very low (one) to very high (five), based on the question used by Knowles [31]. To assess mood, two questions were adapted from the previously validated two-item Patient Health Questionnaire (PHQ-2) [32]. Participants responded ‘yes’ or ‘no’ to having often been bothered by feeling down, depressed or hopeless, and by having little interest or pleasure in doing things (anhedonia) during the previous month.

PA was measured at baseline and after three months using an Axivity AX3 3-Axis Logging Accelerometer® worn continuously on the wrist for seven days. We excluded participants who wore the accelerometer for less than 24 h and adjusted the acceleration output for wear time. Participants were also asked to report their recreational activity, based on the previously validated European Prospective Investigation into Cancer and Nutrition (EPIC)-Norfolk Physical Activity Questionnaire [33] to estimate time typically spent per week during the past three months walking, cycling, gardening, and doing DIY, physical exercise and housework. Total self-reported PA was calculated in addition to vigorous PA (walking, cycling and exercising). If total PA was greater than 100 h per week, responses were recoded as missing since this could be assumed to be an error.

Total plasma carotenoid levels were assessed at baseline and follow-up by blood samples as an objective proxy measure of FVI. In addition, participants were asked how many servings of fruit and vegetables they consume on an average day (none, one, two to three, four, or five or more per day). These data were analysed as whether or not participants who failed to eat five portions per day at baseline reported doing so at follow-up.

### Statistical analyses

All statistical analyses were performed using STATA 14.2. Baseline characteristics were summarised as mean and standard deviation (mean ± SD) or number and percentage of participants, as appropriate. Where data were available at baseline and follow-up, we used paired two-sample t tests, Wilcoxon signed-rank tests or Pearson’s chi-squared tests to test for a significant difference.

We investigated the relationships between social support, stress and mood, and change in PA and FVI between baseline and follow-up using linear regression for continuous outcomes and logistic regression for binary outcomes. Models adjusted for baseline values of the outcome (unadjusted models) and also for age, sex, randomisation group, level of income, level of education, marital status and type of occupation at baseline (adjusted models) are presented. Adjusted models are referred to in the text unless otherwise stated and *p* = 0.05 is used to define statistical significance. Adjusted linear regression models were tested for normal distribution and homoscedasticity of residuals.

We also conducted sensitivity analyses where data below the 5th and above the 95th percentiles were replaced with the values of the 5th and 95th percentiles, respectively, for continuous outcomes. Sensitivity analyses for the total population and also stratified by sex are shown. We used Wilcoxon rank sum tests to test whether there was a difference between those with and without missing data for objective outcomes at either baseline or follow-up, with regards to demographics and exposures.

## Results

### Participants

The main findings from the INFORM trial of the effect of provision of risk information alongside web-based lifestyle advice on objectively measured levels of PA and FVI will be published separately. Table 1 shows the baseline characteristics of the 716 blood donors included in this analysis, which excluded the control arm. 56% were male and the mean age was 57 years. Over half of participants had a university education (57%) and an annual income below £40,000 (63%). 74% were married or cohabiting and 98% classified their ethnicity as white. Most had a sedentary job (52%) although a substantial number reported not working at present (29%). There were similar numbers in each arm of the trial (238 or 239 participants).

**Table 1:** Baseline demographics and psychosocial factors of participants in the INFORM trial

	N	n (%) or mean ± SD
Demographics		
Sex (male)	716	399 (55.7)
Age (years)	716	56.8 ± 8.8
Education (university)	716	408 (57.0)
Income (less than £40,000 per year)	653	409 (62.6)
Married or living as married	716	530 (74.0)
Ethnicity (White – British, Irish or other)	643	630 (98.0)
Occupation category	716	
Sedentary		370 (51.7)
Do not work at present		208 (29.1)
Randomisation group	716	
(1) Lifestyle advice		239 (33.4)
(2) Lifestyle + phenotype		239 (33.4)
(3) Lifestyle + phenotype + genotype		238 (33.2)
Social support		
Overall	693	5.61 ± 1.13
Family	696	5.55 ± 1.34
Friends	697	5.45 ± 1.27
Significant other	694	5.82 ± 1.47
Stress level	710	
Low		296 (41.7)
Moderate		320 (45.1)
High		94 (13.2)
Mood		
Feeling down	710	133 (18.7)
Anhedonia	707	101 (14.3)

Number and percentage of participants are presented for categorical variables; mean and standard deviation are presented for continuous variables

*N/n* number of participants, *SD* standard deviation

Randomisation groups (1) lifestyle advice only; (2) lifestyle advice plus phenotypic CHD risk score information; (3) lifestyle advice plus phenotypic and genetic CHD risk score information

‘Feeling down’ refers to the question “During the past month, have you often been bothered by feeling down, depressed, or hopeless?” ‘Anhedonia’ refers to the question “During the past month, have you often been bothered by having little interest or pleasure in doing things?”

Table 1 also shows that, on average, participants reported perceiving strong social support at baseline; overall mean score was 5.6 ± 1.1 SD (range one to seven). Participants found greatest support in their significant other and least in their friends. Most participants reported a low or moderate level of stress, although 94 participants (13%) were very stressed. 133 participants (19%) were bothered by feeling down, depressed, or hopeless during the past month, and 101 participants (14%) had anhedonia. Both low mood symptoms were reported by 67 participants (9%).

Participants on average had little increase in their objectively measured PA over the study period (Table 2): average acceleration increased from 13.0 ± 5.6 SD to 13.5 ± 6.8 SD mg/min (*p* = 0.738). However, self-reported total PA increased by an average 50 min to 20.3 ± 12.7 SD hours per week (*p* = 0.003), despite average self-reported vigorous PA decreasing over the study period. Objective FVI, measured by plasma carotenoids, decreased between baseline and follow-up from 3.22 ± 1.44 SD μmol/l to 2.74 ± 1.15 SD μmol/l (*p* < 0.001). Conversely, the number of participants reporting that they eat 5 A Day increased from 218 (31%) to 313 (50%, *p* < 0.001), representing 133 people who increased and 19 who decreased consumption and were re-categorised.

**Table 2:** Physical activity and fruit and vegetable intake in the INFORM trial at baseline and follow-up

	Baseline		Follow-up		*P* value
	N	n (%) or mean ± SD	N	n (%) or mean ± SD	
Physical activity					
Accelerometry					
Time wearing accelerometer (h)*	688	159.8 ± 21.6	624	151.2 ± 31.6	< 0.001^†^
Average acceleration (mg/min)	688	13.0 ± 5.6	624	13.5 ± 6.8	0.738^†^
Self-reported					
Total recreational activity (h/w)	712	19.5 ± 13.4	625	20.3 ± 12.7	0.003^‡^
Vigorous recreational activity (h/w)	715	11.0 ± 9.70	714	10.2 ± 9.60	0.362^§^
Fruit and vegetable intake					
Blood test					
Plasma carotenoid level (μmol/l)	670	3.22 ± 1.44	612	2.74 ± 1.15	< 0.001^||^
Self-reported					
Eating 5 A Day	716	218 (30.5)	630	313 (49.7)	< 0.001^¶^

Number and percentage of participants are presented for categorical variables; mean and standard deviation are presented for continuous variables

*Excluding those who wore the accelerometer for less than 24 h

^†^*n* = 604; using Wilcoxon signed-rank test

^‡^*n* = 623; using Wilcoxon signed-rank test

^§^*n* = 713; using Wilcoxon signed-rank test

^||^*n* = 574; using paired t test

^¶^*n* = 630; using Chi-squared test

*H* hours, *h/w* hours per week, *N/n* number of participants, *SD* standard deviation

There were few missing data overall and few significant differences in characteristics of participants with and without missing data for the objective outcomes at either baseline or follow-up (see Additional file 1: Table S1). There were 28 participants (4%) at baseline and 92 participants (13%) at follow-up without accelerometry data plus 46 participants (6%) at baseline and 104 participants (15%) at follow-up without carotenoid measurements. Those missing data were similar in terms of demographics to those with data, except that participants missing objective FVI were younger than those with data (57.1 verses 55.4 years, *p* = 0.045). Participants with missing outcome data tended to perceive lower social support, but this was only statistically significant when considering their significant other (mean score 5.9 verses 5.6 for PA data, *p* = 0.037; 5.9 verses 5.6 for FVI data, *p* = 0.011) and not their overall social support.

### Association between psychosocial factors and change in PA (Table 3)

No association was observed between psychosocial factors and change in objective PA. Conversely, stress and low mood symptoms were negatively associated with change in self-reported PA. Per unit higher level of stress, change in vigorous PA was 1.5 h per week smaller (95% CI -2.3 to -0.8 h per week, *p* < 0.001) and change in total PA was 1.4 h per week smaller (95% CI -2.4 to -0.4 h per week, *p* = 0.006). Anhedonia appeared to have a larger impact than feeling down, with 1.6 h per week less change in vigorous PA reported for participants who had anhedonia compared to 0.7 h less in those who were feeling down (95% CI -3.5 to 0.2, *p* = 0.082 and 95% CI -2.4 to 0.9, *p* = 0.383). However, all of these associations between mood and change in self-reported PA were weaker and not statistically significant when adjusted for potential confounders. These associations tended to remain but were weaker in the sensitivity analysis, with the exception of anhedonia, which was significantly associated with change in objective PA in the opposite direction to that observed for subjective PA and particularly in males (see Additional file 1: Table S2).

**Table 3:** The association between psychosocial factors and change in physical activity outcomes

	Unadjusted			Adjusted		
	N	β (95% CI)	*P* value	N	β (95% CI)	*P* value
Social support						
Objective						
Acceleration (mg/min)	588	-0.37 (-0.83 to 0.09)	0.111	538	-0.29 (-0.83 to 0.24)	0.283
Self-report						
Total physical activity (h/w)	608	-0.27 (-1.02 to 0.48)	0.475	560	0.03 (-0.78 to 0.84)	0.945
Vigorous physical activity (h/w)	690	-0.58 (-1.16 to 0.00)	0.051	631	0.09 (-0.53 to 0.71)	0.778
Stress						
Objective						
Acceleration (mg/min)	599	0.19 (-0.37 to 0.76)	0.501	547	0.09 (-0.58 to 0.76)	0.795
Self-report						
Total physical activity (h/w)	619	-1.89 (-2.82 to -0.96)	< 0.001	569	-1.42 (-2.44 to -0.41)	0.006
Vigorous physical activity (h/w)	707	-1.73 (-2.46 to -1.00)	< 0.001	645	-1.53 (-2.30 to -0.75)	< 0.001
Mood (feeling down)						
Objective						
Acceleration (mg/min)	598	0.08 (-1.22 to 1.38)	0.903	546	-0.06 (-1.49 to 1.37)	0.935
Self-report						
Total physical activity (h/w)	618	-2.40 (-4.54 to -0.26)	0.028	568	-1.34 (-3.53 to 0.85)	0.230
Vigorous physical activity (h/w)	707	-1.33 (-3.01 to 0.35)	0.120	645	-0.74 (-2.42 to 0.93)	0.383
Mood (anhedonia)						
Objective						
Acceleration (mg/min)	597	0.93 (-0.34 to 2.20)	0.152	545	1.03 (-0.36 to 2.41)	0.146
Self-report						
Total physical activity (h/w)	618	-2.43 (-4.84 to -0.03)	0.048	568	-1.49 (-3.95 to 0.97)	0.235
Vigorous physical activity (h/w)	704	-2.16 (-4.02 to -0.29)	0.024	643	-1.64 (-3.50 to 0.21)	0.082

Assessed by multiple linear regression, reporting beta coefficients. Vigorous physical activity includes walking, cycling and sport. All models are adjusted for baseline PA; the adjusted model is also adjusted for age, sex, randomisation group, marital status, income level, education level and occupation type

*95% CI* 95% confidence interval, *h/w* hours per week

Furthermore, Additional file 1: Table S2 shows that there were only minor differences between males and females in associations between psychosocial factors and change in PA overall. An exception is noted: the association between stress and change in reported vigorous PA was much greater in males than in females (-1.2 h per week in males, 95% CI -2.0 to -0.5, *p* = 0.002; -0.3 h per week in females, 95% CI -1.1 to 0.6, *p* = 0.533). In addition, despite uncertainty, low mood tended to have stronger associations with change in PA among females, and social support may have had a more positive association with reported vigorous PA in males.

### Association between psychosocial factors and change in FVI (Table 4)

As for objective PA, no association was observed between psychosocial factors and change in objective FVI levels. No effect of excluding extreme data was observed for change in carotenoids (see Additional file 1: Table S3). Anhedonia was associated with a smaller change in total carotenoid intake in males (0.3 μmol/l less, 95% CI -0.5 to -0.1, *p* = 0.013) but not in females. A similar association was seen in those feeling down, but was not significant.

**Table 4:** The association between psychosocial factors and change in fruit and vegetable intake outcomes

		Unadjusted		Adjusted		
	N	β/OR (95% CI)	*P* value	N	β/OR (95% CI)	*P* value
Social support						
Objective						
Total carotenoids (μmol/l)	562	-0.03 (-0.09 to 0.03)	0.353	515	-0.04 (-0.10 to 0.03)	0.235
Self-report						
Increase to 5 A Day	419	1.39 (1.12 to 1.72)	0.002	391	1.33 (1.05 to 1.69)	0.020
Stress						
Objective						
Total carotenoids (μmol/l)	570	0.02 (-0.05 to 0.09)	0.617	521	0.02 (-0.06 to 0.10)	0.619
Self-report						
Increase to 5 A Day	428	0.55 (0.43 to 0.71)	< 0.001	399	0.57 (0.43 to 0.76)	< 0.001
Mood (feeling down)						
Objective						
Total carotenoids (μmol/l)	569	-0.04 (-0.21 to 0.12)	0.604	520	-0.05 (-0.22 to 0.13)	0.600
Self-report						
Increase to 5 A Day	427	0.56 (0.32 to 0.99)	0.045	398	0.70 (0.38 to 1.28)	0.246
Mood (anhedonia)						
Objective						
Total carotenoids (μmol/l)	567	-0.10 (-0.29 to 0.10)	0.320	519	-0.12 (-0.32 to 0.08)	0.247
Self-report						
Increase to 5 A Day	427	0.44 (0.23 to 0.85)	0.014	398	0.55 (0.28 to 1.11)	0.095

Assessed by multiple linear regression, reporting beta coefficients for objective outcomes; or logistic regression, reporting odds ratios for self-reported outcomes. The linear regression model is adjusted for baseline carotenoid level; adjusted models are also for age, sex, randomisation group, marital status, income level, education level and occupation type

*95% CI* 95% confidence interval, *OR* odds ratio

Large associations were seen when considering change in the reported number of portions of fruit and vegetables consumed. For each unit increase in social support, a 1.3 times greater likelihood of increasing FVI to 5 A Day (95% CI 1.1 to 1.7, *p* = 0.020) was observed. Stratification by sex shows that this was driven by the strong association between higher social support and greater FVI in females (Additional file 1: Table S3). Each unit increase in stress was associated with nearly half the odds of increasing to 5 A Day (OR 0.6, 95% CI 0.4 to 0.8, *p* < 0.001). Again, the association was much stronger in females, who were 0.4 times less likely to eat 5 A Day per unit increase in stress (95% CI 0.2 to 0.6, *p* < 0.001).

Similarly, reporting symptoms of low mood almost halved the odds of reporting increased FVI, and having anhedonia had a stronger association than feeling down. The strength of these associations was reduced by adjusting for potential confounders (OR 0.7, 95% CI 0.4 to 1.3, *p* = 0.246 for feeling down; OR 0.6, 95% CI 0.3 to 1.1, *p* = 0.095 for anhedonia). The association with mood symptoms again tended to be greater in females when subjective outcomes were considered.

## Discussion

To our knowledge, this is the first analysis of the association between psychosocial factors and change in both objective and subjective PA and FVI outcomes. When self-reported behaviour change was considered, consistent evidence was found for the role of stress in reducing behaviour change, some evidence for mood (where experiencing anhedonia had a greater negative effect than feeling down) and some evidence for an association between social support and FVI. Sometimes the association with change in objective outcomes was in the same direction but none were statistically significant. Overall, there tended to be stronger associations between these psychosocial factors and change in self-reported FVI than PA and in females than males.

One of the strengths of this study is the inclusion of both objective and subjective outcomes. The use of objective measures overcomes issues of reporting bias due to recall error and social desirability to report healthy behaviours, giving a more accurate quantification of the association [34, 35]. The self-reported outcomes provide insight into participants’ subjective views of their behaviour, which is more in line with self-monitoring after the study. A further strength is the low loss to follow-up (15% or less) therefore low risk of attrition bias, and that any differences between those with and without missing outcome data were small.

On the other hand, the generalisability of our findings is limited because our study population consisted of research-active blood donors who are known to have better health and higher socioeconomic status than the general population [36, 37]. Baseline psychosocial factors of participants appear to be comparable to those of the general population, while slightly more met the 5 A Day target at baseline and are likely to be more active than the general population [8, 36]. This may explain some of the observed lack of intervention effect because PA and fruit and vegetable consumption may already be a part of participants’ usual lifestyle, which gives them less capacity for improvement and encouragement to change following receipt of CVD risk information and lifestyle advice. Consequently, the associations presented here may be conservative estimates; alternatively, unhealthier populations may be less motivated to change following receipt of risk information and lifestyle advice.

In addition, the study had a short follow-up of three months meaning that only conclusions about the association of psychosocial factors with short term lifestyle behaviour change can be made. Residual confounding by variables not considered in the design or analysis may also explain some of the associations observed and the 95% statistical significance level should be interpreted with caution due to multiple hypotheses being tested. Moreover, a crude, yes/no form of the PHQ-2 was used (it more commonly asks how frequently the two symptoms are experienced) that may have been answered differently depending on whether someone felt depressed while completing the questionnaire. Nevertheless, it is a simple measure to detect the direction of effect and has been found to be comparable to other depression screening questionnaires [38]. It is also important to note that vigorous self-reported PA and plasma carotenoid levels decreased in the whole cohort over the study period, although we are still able to assess the association between psychosocial factors and change in these outcomes (such as whether these factors were associated with a smaller decrease).

Previous studies investigating the association between social support and healthy behaviours have been inconsistent, with some reporting an association [12–15, 18], and others not [16, 17, 19]. These studies have been in similar populations to ours, that is, in high income countries and populations without CVD. The different findings may reflect heterogeneity in the intervention, such as using a nurse-led counselling intervention [16] or the study design [13]. Our study is a simple before and after evaluation of a web-based intervention in a community setting. Furthermore, there is less literature on the role of stress and mood in behaviour change, but our findings are consistent with the what is known about the determinants of behaviour: higher levels of stress lead to less activity and consumption of unhealthy food [20–22, 39]. Similarly, depression is associated with less engagement with healthful behaviour and behaviour change programmes [24, 26, 27].

Many of the associations were only observed in self-reported PA or FVI. The objective models may reflect the reality of very little association between psychosocial factors and lifestyle behaviour change in this cohort. Conversely, the smaller, less heterogeneous objective change in these behaviours could have attenuated associations with psychosocial factors, in comparison to the subjective outcomes. Alternatively, the associations in subjective outcomes could be due to differences in how questionnaires are completed according to level of stress or low mood, affecting accuracy of recall or engagement. For example, it is possible that all participants experienced social desirability pressure and exaggerated their recording of PA and FVI to be healthier, particularly at follow-up, because they knew that the trial aimed to influence behaviour. Both high and low exposure groups would have experienced this social desirability bias, but those experiencing stress or depression may be more inclined to record desirable outcomes in practice. It is also recognised that social desirability bias is stronger in women [40], in line with our finding of stronger associations of psychosocial factors in females.

The effect sizes observed for self-reported outcomes were large, such as 100 min lower change in vigorous PA per week for each unit increase in stress and around half the likelihood of increasing FVI when experiencing stress and low mood. If these reflect true changes in behaviour, these associations are likely to have an observable impact on daily lifestyle, with downstream impacts on CVD risk. Recent meta-analyses show that increasing PA from inactive to meeting PA guidelines is associated with 23% lower CVD mortality [41] and that each additional portion of fruit or vegetables consumed every day is associated with 4% lower CVD mortality [42]. However, the association with psychosocial factors may have been exaggerated by the self-report measures, although it was sometimes supported by consistency in direction of association with objective measures.

While the longer term associations require further research, these findings are important for those developing and evaluating future lifestyle behaviour change interventions. For example, interventions may be more effective if they include additional aspects to encourage perseverance in the programme during stress, enable counselling alongside behaviour change in people with low mood, or highlight the benefits of healthy behaviours on stress and depression. Similarly, women in particular could be encouraged to discuss their plans for increasing FVI with others. The discrepancies observed in this study between objective and subjective outcomes across groups with different psychosocial characteristics also highlight the need to consider the role of these psychosocial factors when planning and interpreting research using subjective measures.

## Conclusion

We have shown that high stress, low mood and lack of social support may reduce reported behaviour change in response to an intervention giving CVD risk information and lifestyle advice, particularly among women. The role of psychosocial factors should be considered when developing, tailoring and evaluating future interventions.

## Additional file

Additional file 1: **Table S1.** Baseline demographics and psychosocial factors of participants in the INFORM trial, by those with missing data for objective outcomes at baseline or follow-up. **Table S2.** The association between psychosocial factors and change in physical activity outcomes for the total population and stratified by sex (sensitivity analysis). **Table S3.** The association between psychosocial factors and change in fruit and vegetable intake outcomes for the total population and stratified by sex (sensitivity analysis). (PDF 549 kb)

## Abbreviations

CHD: Coronary Heart Disease
CI: Confidence Interval
CVD: Cardiovascular Disease
FVI: Fruit and Vegetable Intake
MSPSS: Multidimensional Scale of Perceived Social Support
OR: Odds Ratio
PA: Physical Activity
PHQ-2: Two-item Patient Health Questionnaire
SD: Standard Deviation
