# Supplementary material for: The association between psychosocial factors and change in lifestyle behaviour following lifestyle advice and information about cardiovascular disease risk
Source: BMC Public Health. 2018 Jun 13;18:731. doi: 10.1186/s12889-018-5655-7 (PMC6001007; doi:10.1186/s12889-018-5655-7)
Supplement: Supplementary file 1 — Table S1. Baseline demographics and psychosocial factors of participants in the INFORM trial, by those with missing data for objective outcomes at baseline or follow-up. Table S2. The association between psychosocial factors and change in physical activity outcomes for the total population and stratified by sex (sensitivity analysis). Table S3. The association between psychosocial factors and change in fruit and vegetable intake outcomes for the total population and stratified by sex (sensitivity analysis). (PDF 549 kb) [file 12889_2018_5655_MOESM1_ESM.pdf]

Additional File 1

Table S1: Baseline demographics and psychosocial factors of participants in the INFORM trial, by those with missing data for objective outcomes at baseline or follow-up.

	Objective physical activity					Objective fruit and vegetable intake				
	Non-missing		Missing			Non-missing		Missing		
	N	n (%) or mean ± SD	N	n (%) or mean ± SD	P value	N	n (%) or mean ± SD	N	n (%) or mean ± SD	P value
**Demographics**										
Sex (male)	604	332 (55.0)	112	67 (59.8)	0.343	574	315 (54.9)	142	84 (59.2)	0.359
Age (years)	604	56.8 ± 8.9	112	56.6 ± 8.7	0.817	574	57.1 ± 8.73	142	55.4 ± 9.15	0.045
Education (university)	604	338 (56.0)	112	70 (62.5)	0.200	574	329 (57.3)	142	79 (55.6)	0.717
Income (less than £40 000 per year)	552	352 (63.8)	101	57 (65.4)	0.162	525	333 (63.4)	128	76 (59.4)	0.396
Married or living as married	604	453 (75.0)	112	77 (68.8)	0.166	574	432 (75.3)	142	98 (69.0)	0.129
Ethnicity (White – British, Irish or other)	541	531 (98.2)	102	99 (97.1)	0.472	512	503 (98.2)	131	127 (97.0)	0.348
Randomisation group	604		112			574		142		
(1) Lifestyle advice		200 (33.1)		39 (34.8)	0.787		196 (34.2)		43 (30.3)	0.242
(2) Lifestyle + phenotype		207 (34.3)		32 (28.6)			193 (33.6)		46 (32.4)	
(3) Lifestyle + phenotype + genotype		197 (32.6)		41 (36.6)			185 (32.2)		53 (37.3)	
Occupation category	604		112			574		142		
Sedentary		306 (50.7)		64 (57.1)	0.246		290 (50.5)		80 (56.3)	0.146
Do not work at present		179 (29.6)		29 (25.9)			174 (30.3)		34 (23.9)	
**Social support**										
Overall	588	5.63 ± 1.12	105	5.48 ± 1.18	0.184	562	5.64 ± 1.13	131	5.48 ± 1.14	0.109
Family	591	5.57 ± 1.33	105	5.44 ± 1.41	0.392	564	5.57 ± 1.36	132	5.44 ± 1.29	0.128
Friends	592	5.46 ± 1.27	105	5.39 ± 1.29	0.480	564	5.47 ± 1.26	133	5.34 ± 1.32	0.294
Significant other	589	5.86 ± 1.47	105	5.60 ± 1.46	0.037	562	5.87 ± 1.47	132	5.64 ± 1.45	0.011
**Stress level**	599		111			570		140		
Low		258 (43.1)		38 (34.2)	0.225		249 (43.7)		47 (33.6)	0.070
Moderate		260 (43.4)		60 (54.1)			246 (43.2)		74 (52.9)	
High		81 (13.5)		13 (11.7)			75 (13.2)		19 (13.6)	
**Mood**										
Feeling down	598	113 (18.9)	112	20 (17.9)	0.796	569	109 (19.2)	141	24 (17.0)	0.561
Anhedonia	597	88 (14.7)	110	13 (11.8)	0.421	567	75 (13.2)	140	26 (18.6)	0.106

Number and percentage of participants are presented for categorical variables; mean and standard deviation are presented for continuous variables.

N/n number of participants; **SD** standard deviation

Randomisation groups (1) lifestyle advice only; **(2)** lifestyle advice plus phenotypic CHD risk score information; **(3)** lifestyle advice plus phenotypic and genetic CHD risk score information

'Feeling down' refers to the question "During the past month, have you often been bothered by feeling down, depressed, or hopeless?" 'Anhedonia' refers to the question "During the past month, have you often been bothered by having little interest or pleasure in doing things?"

Additional File 1

Supplementary Table 2: The association between psychosocial factors and change in physical activity outcomes for the total population and stratified by sex (sensitivity analysis).

	All						Male						Female					
	Unadjusted			Adjusted			Unadjusted			Adjusted			Unadjusted			Adjusted		
	N	β (95% CI)	P value	N	β (95% CI)	P value	N	β (95% CI)	P value	N	β (95% CI)	P value	N	β (95% CI)	P value	N	β (95% CI)	P value
**Social support**																		
**Objective**																		
Acceleration (mg/min)	588	-0.24 (-0.52 to 0.05)	0.100	538	-0.21 (-0.53 to 0.12)	0.214	323	-0.29 (-0.68 to 0.10)	0.150	302	-0.25 (-0.71 to 0.21)	0.288	265	-0.19 (-0.60 to 0.23)	0.382	236	-0.14 (-0.62 to 0.33)	0.548
**Self-report**																		
Total physical activity (h/w)	608	0.07 (-0.52 to 0.65)	0.827	560	0.24 (-0.39 to 0.87)	0.458	333	-0.29 (-1.07 to 0.50)	0.472	314	0.49 (-0.39 to 1.38)	0.274	275	0.40 (-0.50 to 1.30)	0.382	246	0.38 (-0.55 to 1.32)	0.421
Vigorous physical activity (h/w)	690	-0.05 (-0.46 to 0.35)	0.791	631	0.17 (-0.28 to 0.62)	0.468	386	0.09 (-0.48 to 0.65)	0.767	361	0.46 (-0.20 to 1.11)	0.172	304	-0.30 (-0.88 to 0.28)	0.304	270	-0.03 (-0.65 to 0.59)	0.928
**Stress**																		
**Objective**																		
Acceleration (mg/min)	599	0.19 (-0.16 to 0.54)	0.297	547	0.22 (-0.19 to 0.63)	0.291	331	0.21 (-0.26 to 0.68)	0.377	309	0.28 (-0.27 to 0.82)	0.316	268	0.15 (-0.39 to 0.69)	0.587	238	0.12 (-0.51 to 0.76)	0.699
**Self-report**																		
Total physical activity (h/w)	619	-1.27 (-1.99 to -0.55)	0.001	569	-0.88 (-1.67 to -0.10)	0.027	340	-1.19 (-2.10 to -0.28)	0.010	320	-0.88 (-1.86 to 0.10)	0.078	279	-1.44 (-2.61 to -0.27)	0.016	249	-0.84 (-2.12 to 0.44)	0.197
Vigorous physical activity (h/w)	707	-0.86 (-1.37 to -0.35)	0.001	645	-0.82 (-1.39 to -0.25)	0.005	396	-1.19 (-1.88 to -0.51)	0.001	369	-1.23 (-1.99 to -0.46)	0.002	311	-0.40 (-1.16 to 0.36)	0.305	276	-0.27 (-1.11 to 0.58)	0.533
**Mood (feeling down)**																		
**Objective**																		
Acceleration (mg/min)	598	-0.29 (-1.09 to 0.52)	0.484	546	-0.29 (-1.16 to 0.57)	0.507	329	0.12 (-1.03 to 1.28)	0.832	307	0.06 (-1.15 to 1.28)	0.919	269	-0.76 (-1.88 to 0.36)	0.184	239	-0.78 (-2.03 to 0.48)	0.222
**Self-report**																		
Total physical activity (h/w)	618	-1.69 (-3.34 to -0.04)	0.044	568	-0.90 (-2.58 to 0.78)	0.293	338	-1.15 (-3.32 to 1.03)	0.301	318	-0.61 (-2.81 to 1.58)	0.582	280	-2.36 (-4.89 to 0.17)	0.067	250	-1.39 (-4.02 to 1.24)	0.298
Vigorous physical activity (h/w)	707	-0.80 (-1.96 to 0.37)	0.179	645	-0.45 (-1.66 to 0.76)	0.465	394	-0.93 (-2.59 to 0.72)	0.268	367	-0.30 (-2.02 to 1.42)	0.734	313	-0.72 (-2.33 to 0.90)	0.383	278	-0.64 (-2.34 to 1.06)	0.459
**Mood (anhedonia)**																		
**Objective**																		
Acceleration (mg/min)	597	0.81 (-0.07 to 1.68)	0.071	545	1.01 (0.07 to 1.96)	0.035	329	1.01 (-0.12 to 2.14)	0.079	307	1.12 (-0.08 to 2.31)	0.066	268	0.51 (-0.92 to 1.93)	0.484	238	0.42 (-1.16 to 2.00)	0.601
**Self-report**																		
Total physical activity (h/w)	618	-1.58 (-3.44 to 0.28)	0.095	568	-0.88 (-2.78 to 1.01)	0.361	338	-1.14 (-3.36 to 1.08)	0.313	318	-0.90 (-3.18 to 1.38)	0.439	280	-2.11 (-5.40 to 1.19)	0.209	250	-1.90 (-5.27 to 1.47)	0.268
Vigorous physical activity (h/w)	704	-1.31 (-2.60 to -0.02)	0.046	643	-0.93 (-2.27 to 0.42)	0.177	394	-1.09 (-2.78 to 0.60)	0.206	367	-0.75 (-2.52 to 1.01)	0.402	310	-1.66 (-3.70 to 0.39)	0.112	276	-1.86 (-3.97 to 0.26)	0.085

Assessed by multiple linear regression, reporting beta coefficients. Vigorous physical activity includes walking, cycling and sport. Sensitivity analysis: data below 5th/above 95th percentiles replaced with the values of the 5th/95th percentiles, respectively. All models are adjusted for baseline PA; the adjusted model is also adjusted for age, sex, randomisation group, marital status, income level, education level and occupation type.

95% CI 95% confidence interval; **h/w** hours per week; **N/n** number of participants

Additional File 1

Table S3: The association between psychosocial factors and change in fruit and vegetable intake outcomes for the total population and stratified by sex (sensitivity analysis).

	All						Males						Females					
	Unadjusted			Adjusted			Unadjusted			Adjusted			Unadjusted			Adjusted		
	N	β/OR (95% CI)	P value	N	β/OR (95% CI)	P value	N	β/OR (95% CI)	P value	N	β/OR (95% CI)	P value	N	β/OR (95% CI)	P value	N	β/OR (95% CI)	P value
**Social support**																		
**Objective**																		
Total carotenoids (µmol/l)	562	-0.02 (-0.07 to 0.03)	0.481	515	-0.03 (-0.09 to 0.03)	0.289	311	-0.00 (-0.07 to 0.06)	0.925	293	-0.02 (-0.09 to 0.06)	0.659	251	-0.03 (-0.11 to 0.06)	0.493	222	-0.04 (-0.13 to 0.06)	0.447
**Self-report**																		
Increase to 5 A Day	419	1.39 (1.12 to 1.72)	0.002	391	1.33 (1.05 to 1.69)	0.020	246	1.27 (0.96 to 1.68)	0.097	229	1.19 (0.85 to 1.66)	0.303	173	1.56 (1.12 to 2.18)	0.009	157	1.68 (1.12 to 2.53)	0.012
**Stress**																		
**Objective**																		
Total carotenoids (µmol/l)	570	0.01 (-0.05 to 0.07)	0.728	521	0.01 (-0.06 to 0.08)	0.805	315	-0.00 (-0.07 to 0.07)	0.969	296	-0.02 (-0.11 to 0.06)	0.602	255	0.03 (-0.08 to 0.14)	0.634	225	0.04 (-0.09 to 0.17)	0.539
**Self-report**																		
Increase to 5 A Day	428	0.55 (0.43 to 0.71)	<0.001	399	0.57 (0.43 to 0.76)	<0.001	251	0.66 (0.49 to 0.89)	0.006	234	0.67 (0.47 to 0.96)	0.028	177	0.38 (0.24 to 0.59)	<0.001	160	0.35 (0.20 to 0.62)	<0.001
**Mood (feeling down)**																		
**Objective**																		
Total carotenoids (µmol/l)	569	-0.04 (-0.19 to 0.11)	0.592	520	-0.04 (-0.20 to 0.11)	0.586	313	-0.11 (-0.29 to 0.07)	0.234	294	-0.14 (-0.33 to 0.05)	0.157	256	0.02 (-0.22 to 0.26)	0.870	226	0.03 (-0.23 to 0.30)	0.796
**Self-report**																		
Increase to 5 A Day	427	0.56 (0.32 to 0.99)	0.045	398	0.70 (0.38 to 1.28)	0.246	250	0.70 (0.33 to 1.52)	0.369	233	0.84 (0.37 to 1.93)	0.683	177	0.42 (0.18 to 0.97)	0.043	160	0.55 (0.22 to 1.39)	0.208
**Mood (anhedonia)**																		
**Objective**																		
Total carotenoids (µmol/l)	567	-0.12 (-0.29 to 0.05)	0.173	519	-0.15 (-0.33 to 0.03)	0.109	314	-0.20 (-0.39 to -0.00)	0.046	295	-0.26 (-0.46 to -0.06)	0.013	253	-0.01 (-0.31 to 0.30)	0.958	224	-0.04 (-0.37 to 0.29)	0.803
**Self-report**																		
Increase to 5 A Day	427	0.44 (0.23 to 0.85)	0.014	398	0.55 (0.28 to 1.11)	0.095	249	0.54 (0.25 to 1.19)	0.125	232	0.68 (0.28 to 1.61)	0.375	178	0.29 (0.08 to 1.02)	0.054	161	0.31 (0.08 to 1.14)	0.078

Assessed by multiple linear regression, reporting beta coefficients for objective outcomes; or logistic regression, reporting odds ratios for self-reported outcomes. Sensitivity analysis: data below 5th/above 95th percentiles replaced with the values of the 5th/95th percentiles, respectively, for continuous outcomes. The linear regression model is adjusted for baseline carotenoid level; adjusted models are also for age, sex, randomisation group, marital status, income level, education level and occupation type.

95% CI 95% confidence interval; **OR** odds ratio; **N/n** number of participants
